# Multiscale Free Energy Analysis of Human Ecosystem Engineering

**DOI:** 10.3390/e23040396

**Published:** 2021-03-26

**Authors:** Stephen Fox

**Affiliations:** VTT Technical Research Centre of Finland, FI-02150 Espoo, Finland; stephen.fox@vtt.fi; Tel.: +358-40-747-8801

**Keywords:** active inference, free energy principle, human ecosystem engineering, multiscale free energy, preferences, surprise, survival

## Abstract

Unlike ecosystem engineering by other living things, which brings a relatively limited range of sensations that are connected to a few enduring survival preferences, human ecosystem engineering brings an increasing variety and frequency of novel sensations. Many of these novel sensations can quickly become preferences as they indicate that human life will be less strenuous and more stimulating. Furthermore, they can soon become addictive. By contrast, unwanted surprise from these novel sensations may become apparent decades later. This recognition can come after the survival of millions of humans and other species has been undermined. In this paper, it is explained that, while multiscale free energy provides a useful hypothesis for framing human ecosystem engineering, disconnects between preferences and survival from human ecosystem engineering limit the application of current assumptions that underlie continuous state-space and discrete state-space modelling of active inference.

## 1. Introduction

The free-energy principle (FEP) formalizes embodied cognition of the autopoietic organization of living things. In particular, formalizes that active systems must occupy a limited repertoire of internal states through minimizing the long-term average of unwanted surprise from external states. For example, a flying fish minimizes unwanted surprise through a limited repertoire of internal states. These include being out of water when that best facilitates survival by avoiding underwater predators, and being in water at other times when that best facilitates survival. Evolving a limited repertoire of internal states through iterations of minimizing unwanted surprise can lead to reduction in information entropy arising from external states. In other words, iterations of minimizing unwanted surprise leads to low uncertainty about what sensory inputs will come from external states.

In particular, within FEP, living things have models of what sensory inputs to expect from external states. These are generative models that generate predictions about what will happen. Predictive generative models do not need to involve conscious thought and psychological surprise. Rather, generative models are embodied models that involve body-wide neurology and encompass autonomic behavior. Whether or not generative models involve conscious thought, unwanted surprise arises from a living thing’s generative model of what sensory inputs are preferred to come from external states. Specifically, unwanted surprise arises when what is preferred to happen does not happen. For example, within FEP, flying fish prefer to fly out of dangerous water into safe air and then descend into safe water. Flying fish do not prefer to fly out of water onto the deck of a fishing boat. Rather, flying fish need to minimize long-term average unwanted surprise that can be caused by flying into danger that threatens survival [1,2,3].

FEP posits that whatever the living thing, minimizing unwanted surprise will cause the effect of surviving within preferred internal non-equilibrium steady-state (NESS). Multiscale FEP is a hypothesis that there is an interdependent necessity for life at every scale, for example from sub-cellular to sociocultural, to minimize surprise that threatens survival [4,5]. However, FEP and its corollary active inference theory (AIT) are based on living things that have evolved through many millennia to survive within a few types of natural environments that change little from one generation to the next. Although human embodied cognition also evolved through many millennia surviving in similar environments by avoiding a relatively limited range of unwanted surprises [6], we are now trying to survive in unnatural environments brought by human ecosystem engineering that can change many times within one generation [7]. Notably, unlike ecosystem engineering by other species, which brings a relatively limited range of sensations that are connected to a few enduring survival objectives, human ecosystem engineering brings an increasing variety and frequency of novel sensations [8]. Crucially, many of these novel sensations can quickly become preferences because they indicate that human life will be less strenuous and more stimulating. Furthermore, they can soon become addictive [9]. By contrast, unwanted surprise from these novel sensations may become apparent decades later. This recognition can come after the survival of millions of humans and other species has been undermined [10,11,12,13,14,15].

The purpose of this paper is relate this human disconnect between preferences and survival to multiscale FEP and to different models of active inference. In order to fulfil this purpose, the remainder of the paper proceeds in three further sections. In Section 2, disconnects between preferences and survival are explained through reference to practical examples. In Section 3, they are related to multiscale FEP, continuous state-space models (CSSM), and discrete state-space models (DSSM) of active inference. In the concluding Section 4, principal contributions are stated and directions for future research are proposed.

Overall, this paper demonstrates the potential for FEP to facilitate novel conceptual analysis [16]. Furthermore, this paper demonstrates the potential of FEP to bring together phenomena that have hitherto been investigated separately [13]: here, active inference and technology in society [17,18].

## 2. Disconnects between Preferences and Survival

Ecosystem engineering by other species brings a relatively limited range of sensations that are connected to a few enduring survival imperatives. For example, beavers prefer to observe that there are ponds close-by that can provide them with protection from predators. If they cannot observe ponds close-by, beavers will undertake ecosystem engineering that includes building ponds that will provide them with protection from predators [19]. From the point of view of FEP, connections between preferences, unwanted surprise and survival have evolved to be straightforward. For example, when a beaver makes an observation of a predator, such as a lynx, blocking its path to a protective pond, the beaver makes an observation of an unwanted surprise in the external state that threatens the survival of its internal NESS.

By contrast, connections between preferences, unwanted surprise and survival are far more complex for humans in the 21st century. In many cases, human ecosystem engineering involves human organizations making massive capital investments: the costs of which can only be covered if there is widespread use. For example, establishing infrastructures for mass agri-food production, distribution and retailing involves massive capital investment. In order to encourage use, marketing messages are sent to provide novel sensations that increase awareness, interest, desire, and involvement [20].

Typically, marketing messages do not involve emphasizing potential threats. Moreover, threats may not even be known until after many decades of use. For example, the marketing of processed food has highlighted specific attributes to specific market segments on top of the general convenience of saving energy and time that would otherwise be spent preparing food [21]. Processed food has not been marketed on the basis of causing adverse health outcomes, and the unwanted surprise of adverse health outcomes may not have been known until after decades of convenience food mass consumption had begun [22]. Meanwhile, rather than seem threatening, processed foods seem consistent with the primary evolutionary trend towards survival through least action [23], and can stimulate the reward system [24,25]. Thus, processed food is an example of new threats to human survival coming from human ecosystem engineering intended to make human life less strenuous and more stimulating. Similarly, automotive vehicles and their associated infrastructure can make human life less strenuous and more stimulating [26]. Moreover, automotive vehicles replaced horse-drawn vehicles that had caused health threats from emissions of vast quantities of dung [27]. It was not until after many decades that the far more difficult to control threat caused by automotive vehicle emissions was recognized [28].

**Figure 1:**
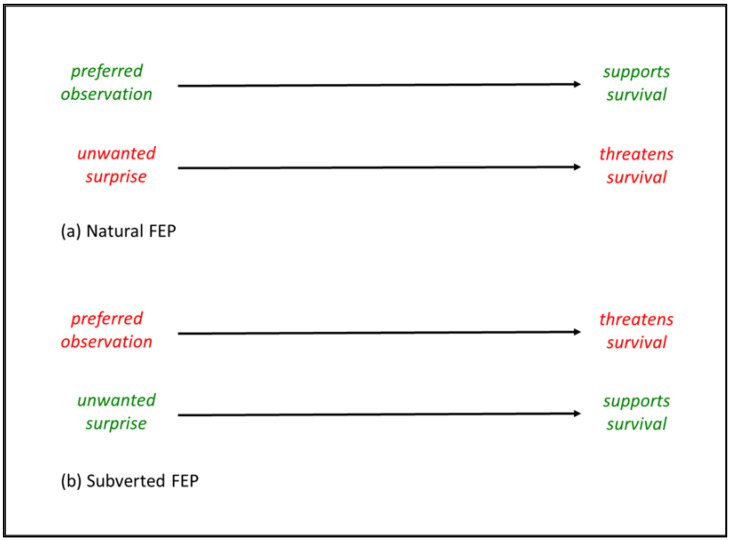
Comparison of (**a**) natural FEP and (**b**) subverted FEP.

Thus, for humans in the 21st century there are many scenarios where there is not the natural connection between preferred observation, unwanted surprise, and survival summarized in Figure 1a. Rather, as summarized in Figure 1b, there can be many scenarios where FEP is subverted because humans’ preferred observations, for example of less strenuous and more stimulating life, are not consistent with survival of internal NESS. By contrast, the eventual unwanted surprise of scientific findings, which indicate that less strenuous and more stimulating has adverse effects, can support survival.

## 3. Implications

### 3.1. Multiscale FEP

Multiscale FEP is a hypothesis that there is an interdependent necessity for life at every scale to minimize surprise that threatens survival. However, as summarized in Figure 1, human ecosystem engineering introduces disconnects between unwanted surprise and survival in the form of often alluring and sometimes addictive opportunities for human life to be less strenuous and more stimulating. These opportunities often to lead to many decades of humans’ preferred observation, rather than unwanted surprise, being connected to threats to survival.

This does not mean that the multiscale FEP hypothesis is incorrect. Rather, the multiscale FEP hypothesis is supported by the human trend to eventually identify and address causes of preferred observations threatening survival. Apropos, cycles of human ecosystem engineering and humans rectifying consequent threats to survival can be seen as cycles of biosocio-technical evolution to meet FEP. Furthermore, the threat to human survival arising from damage done to biodiversity by human ecosystem engineering highlights interdependencies between different scales of life. In particular, human subversion of FEP causes chaotic disruptions down to the microbial level and up to the planetary level, which in turn brings unwanted surprises that threaten human survival [29,30].

### 3.2. Continuous State-Space Modeling (CSSM)

CSSM of active inference involves modelling autopoietic organization as continuously evolving random dynamical system for minimizing surprise. This involves conditional dependencies between internal states and external states, within which internal states have generative models about external states. In particular, the internal state parameterizes a probability distribution over the external states, which maps the causes of sensory inputs from external states. Hence, generative models can be considered as a joint probability distribution over internal states and external states [31,32]. However, formulating joint probability distribution over internal states and external states may not be possible when there is lack of consensus about what is going on in external states.

For example, automotive vehicles can make human life less strenuous and more stimulating but perhaps threaten survival by contributing to climate change. However, they are not the only contributor, and agreeing the relative contribution of different factors in different cases may be almost impossible [33]. Moreover, climate change is not consistent over time and/or at locations. For example, nations that have high levels of greenhouse gas emissions may experience little climate change, while nations that have low levels of greenhouse gas emissions may experience much climate change. At the same time, some who gain local advantages may regard climate change as not being a threat to survival [34].

Thus, for humans in the 21st century, formulating joint probability distribution over internal states and external states for CSSM is not necessarily possible. Especially, when rather than there being agreement about threats to survival, there can be on-going campaigns between competing narratives that are rooted in the underlying perspectives of different sociocultural groups [35]. This can involve one side arguing that a type of ecosystem engineering is essential to survival [36], while the other side argues that it is a threat to survival [37]. Thus, instead of formulating joint probability distribution based on scientific evidence about threats to survival, individuals can align with probability distribution preferred by the sociocultural group within which they prefer to attempt to survive in, even if that does not support survival of humanity as a whole. In other words, CSSM should not be predicated on assumptions that generative models, which are a joint probability distribution over internal states and external states, will support survival. This is because they can be based on human preconceptions about the world rather than what is happening in the world. Moreover, these preconceptions can be highly resistant to updating even if there are many incoming sensory signals that are not in accordance with preconceptions. In particular, rather than minimizing unwanted surprise by updating generative models or by changing actions, unwanted surprise is minimized through reinforcing preconceptions, for example, via common phenomena such as wishful seeing and motivated cognition [38,39].

### 3.3. Discrete State-Space Modelling (DSSM)

DSSM of active inference involves modelling autopoietic organization as selection from discrete preferences for minimizing surprise. For example, beavers could have the following preference distribution for sensory inputs: +10 (protective pond); 0 (woodland); −10 (hungry predator). Such a natural preference distribution would evolve through many millennia to facilitate survive within a few types of natural environments that change little from one generation to the next. Within FEP, it can be argued that long-term survival depends upon generative models for preferences having enough time to adapt to environmental changes. For example, within FEP, following human water pollution [40] beavers could develop a new generative model with new preference distribution for sensory inputs: +10 (protective pond in clear water); +2 (protective pond in polluted water); 0 (woodland); −10 (hungry predator).

For humans, eventually after decades of scientific research, there can be consensus information about discrete alternatives for preferred observations to support survival. However, the existence of consensus information does not necessarily lead to humans’ preferred observations being those that would support survival. This is because preferred observations for supporting survival can involve resisting strong temptations to make life less strenuous and more stimulating. Moreover, selecting preferred observations for supporting survival can involve overcoming addictions, which are tangled up with variables that cannot easily be changed, including personality type [41,42,43,44].

Indeed, selecting preferred observations for supporting survival can be extremely difficult even when there is immediate physical evidence of the threats to survival from not doing so. Consider, for example, the immediate physical evidence of obesity, diabetes, and associated amputations that is prevalent in some Pacific Islands, which is linked to abandoning traditional diets in favor of imported processed food. It has been argued that traditional foods cannot compete with the glamour and flashiness of imported foods: even though the dire health consequences of calorie-rich and nutrient-poor imported foods are clearly visible. Ignoring information about preferred observations for survival can start young. For example, schools teach good nutrition but sell junk food in the school canteen because they need to make a profit [45,46,47]. This example illustrates that DSSM should not be automatically predicated on assumptions that humans have preferences that support survival. For example, a preference distribution could be +10 (imported food); +2 (local food); −10 (no food): even though consumption of imported food is clearly not compatible with survival.

## 4. Conclusions

The principal contribution of this paper is to relate FEP to human ecosystem engineering that brings disconnects between preferences and survival. Implications from these disconnects have been described for multiscale FEP, continuous state-space (CSSM) and discrete state-space (DSSM) models of active inference. These introduce several directions for further research. From the point of view of practice [48], FEP can be related to the process of human ecosystem engineering. In particular, a major design principle for human ecosystem engineering can be design for preferences that support survival. Rather than design for life that is less strenuous and more stimulating but undermines survival. Additionally, the potential for CSSM and DSSM to support human ecosystem engineering can be investigated. However, first theoretical developments are needed to enable AIT to encompass disconnects between preferred observations, unwanted surprise, and survival.
